# Soft tissue metastasis revealing a case of acinar cell carcinoma of unknown primary origin

**DOI:** 10.1002/ccr3.3266

**Published:** 2020-08-27

**Authors:** Werda Ines, Belaid Imtinene, Mestiri Sarra, Bedioui Ahlem, Ezzaairi Faten, Chabchoub Imene, Ammar Nouha, Hochlaf Makrem, Ben Fatma Leila, Mokni Moncef, Ben Ahmed Slim

**Affiliations:** ^1^ Faculty of Medicine of Sousse Department of Medical Oncology Farhat Hached University Hospital University of Sousse Sousse Tunisia; ^2^ Faculty of Medicine of Sousse Department of Pathology Farhat Hached University Hospital University of Sousse Sousse Tunisia

**Keywords:** chemotherapy, metastatic acinic cell carcinoma, unknown primitive tumor

## Abstract

Acinic cell carcinoma (ACC) is a rare neoplasm. It can be fatal in some cases and conventional chemotherapy may not be effective. To our knowledge, we report here the first case of ACC metastatic to soft tissues, from unknown origin.

## Graphical abstract

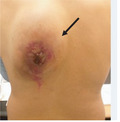

## 1 INTRODUCTION

Acinic cell carcinoma (ACC) is a rare neoplasm that occurs most often in the salivary glands and more rarely in the pancreas and in the lung. Soft tissue metastases from ACC are extremely rare. We report a case of soft tissue metastasis of ACC of unknown origin. A 67‐year‐old man was evaluated with a huge dorsal mass, opposite the left shoulder, of 25*22 cm, neglected for 2 years. MRI of the back showed a tumor which could be have likely features of malignancy, within the left para‐vertebral muscles and invading the left 5th and 6th ribs' posterior arch. A chest CT scan revealed multiple pulmonary metastases. A biopsy was performed. Histological and immunohistochemical examination of the biopsied tumor had revealed soft tissue metastases from ACC. An abdominal CT scan was performed and did not reveal any primary pancreatic lesion or other metastatic lesions. MRI of face and neck, to search for a salivary gland origin of the metastases, was normal. Chemotherapy with Paclitaxel and Carboplatin combination was offered. After three cycles, the patient experienced disease progression. A second line of chemotherapy with FEC (5‐fluorouracil, epirubicin, and cyclophosphamide) was administered. A locoregional and metastatic pulmonary progression was diagnosed after four cycles, and the patient died 6 weeks later. To our knowledge, this is the first reported case of ACC metastatic to soft tissues, of unknown origin. Chemotherapy is the mainstay of the treatment of these metastatic malignancies and the protocol depends on the nature of the primary tumor. In our case, given the unknown primitive, various chemotherapy protocols were administered but were all ineffective. Further studies are needed in order to codify the treatment of these rare entities.

Acinic cell carcinoma is a rare neoplasm that occurs most often in the salivary glands and more rarely in the pancreas and in the lung. It represents 17% of all salivary gland carcinomas, only 2%‐4% of neoplasms of the parotid gland^1^ and about 1% of exocrine pancreatic tumors.^2^ Only 25 cases of primary pulmonary ACC have been reported in the literature.^3^ Soft tissue metastases from ACC are extremely rare.^4^ To the best of our knowledge, it is the first case of soft tissue metastasis of ACC from unknown primary origin published in the literature.

## 2 CASE REPORT

A 67‐year‐old man was referred to our hospital with a left dorsal lump, neglected for 2 years but having recently increased in size.

**FIGURE 1:**
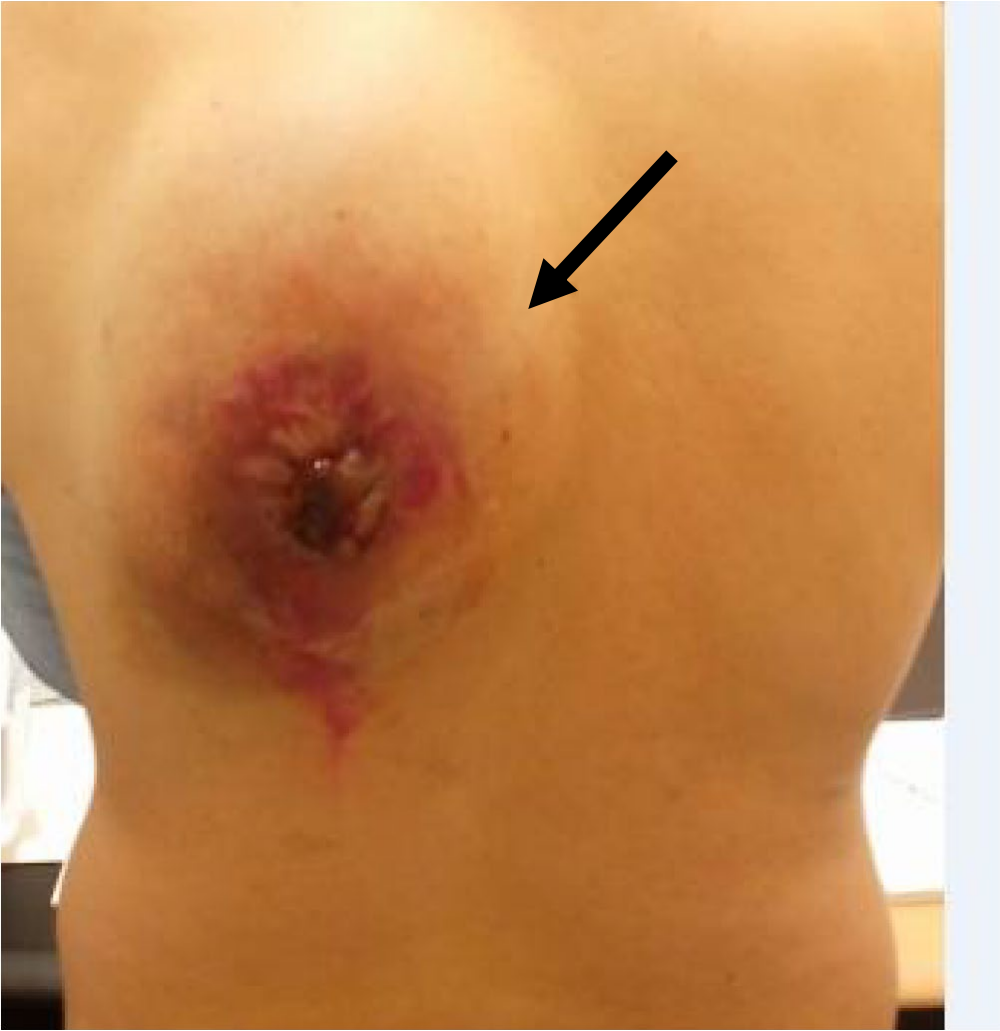
A huge dorsal mass of 25*22 cm with ulcero‐necrotic center, fistulized to the skin, opposite the left shoulder blade

Clinical examination identified a huge dorsal mass of 25*22 cm. It was located opposite the left shoulder blade and it was moderately firm with ulcero‐necrotic center fistulized to the skin, (Figure 1). The patient had no further complaints.

**FIGURE 2:**
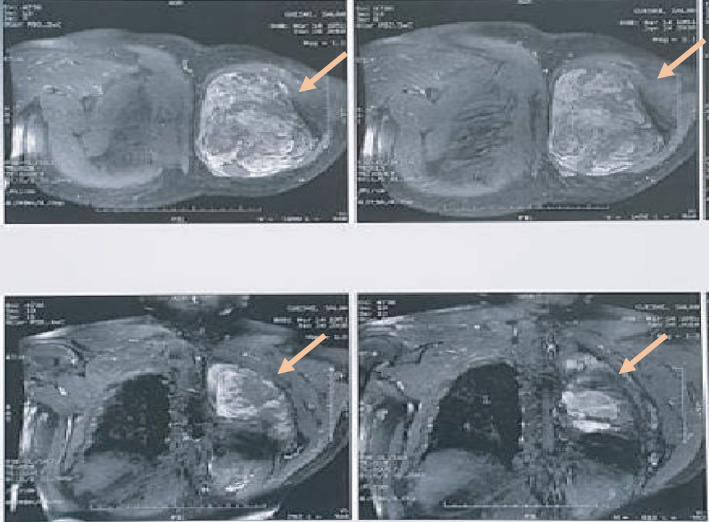
MRI T1 saturated postcontrast (gadolinium) sequence of the dorsal soft tissues showing a malignant tumor of 18.5*20 cm within the left para‐vertebral muscles and invading the posterior arch of the 5th and 6th ribs

**FIGURE 3:**
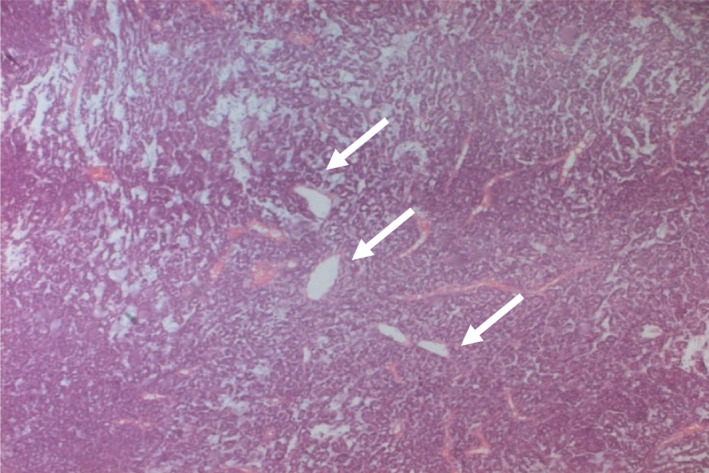
H.E × 40: Ill‐defined malignant epithelial proliferation, with an acinar, lobular pattern, displaying an organoid pattern

**FIGURE 4:**
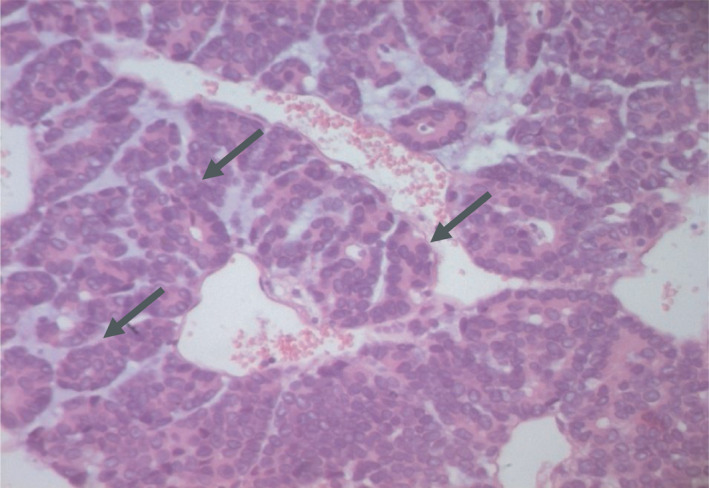
H.E × 200: Tumor cells are monotonous with polarized nuclei and an abundant eosinophilic granular cytoplasm

**FIGURE 5:**
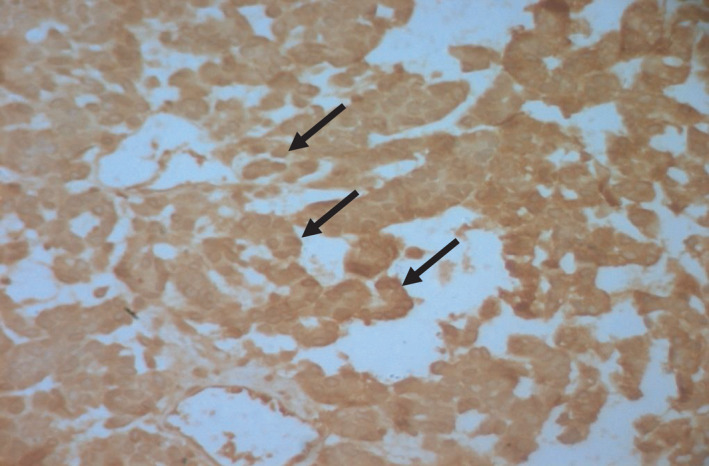
IHC × 200: Positive diffuse and intense immunostaining of tumoral cells with Trypsine

MRI of the dorsal soft tissues showed a malignant tumor of 18.5*20 cm within the left para‐vertebral muscles and invading the 5th and 6th ribs' posterior arch (Figure 2). A chest CT scan revealed multiple pulmonary metastases. A biopsy was performed. Histology identified (Figures 3 and 4) epithelial malignant tumor proliferation, with high cell density and monotonic tumor cells arranged in compact trabeculae or acini, with polarized nuclei arranged radially around acinar lumens; and an abundant eosinophilic granular cytoplasm. Immunochemistry showed an intense and diffuse positivity of tumor cells for alpha‐antitrypsin (acinic marker; Figure 5), a moderate and heterogeneous staining with CD56 (neuroendocrine marker). Other markers such as synaptophysin, chromogranin A, CK19, CK7 were negative. Soft tissue metastasis from an ACC was confirmed.

An abdominal CT scan was performed and did not reveal any pancreatic lesion. MRI of face and neck to search for a salivary gland origin of the metastases was unremarkable.

Chemotherapy with Paclitaxel (175 mg/m^2^ every 3 weeks) and Carboplatin (AUC:5 according to Calvert formula, every 3 weeks) was indicated. After three cycles, the patient experienced disease progression. A second line of chemotherapy by FEC75 (5‐fluorouracil at 500 mg/m^2^ [at day1], epirubicin 75 mg/m^2^ [at day1], Cyclophosphamide 500 mg/m^2^ [at day1], every 3 weeks) was administered. A locoregional and metastatic pulmonary progression were diagnosed after four cycles. The patient died 6 weeks later.

## 3 DISCUSSION

Acinic cell carcinoma is a low‐grade malignancy.^4^ The salivary glands particularly the parotid gland represent the predominant site of origin.^4^ Other areas of primary ACCs include the pancreas, lungs, stomach, breast, and prostate.^5^ Pathologically, conventional ACC is a low‐grade epithelial tumor with at least focal serous acinar differentiation and typically exhibits a microcystic, solid, papillary, or follicular growth pattern.^6^ ACC is a slow‐growing malignant tumor.^7^ A rapid progression of ACC could suggest a dedifferentiation, or high‐grade transformation of this tumor.^7^, ^8^ Contrary to the well‐differentiated type, ACC transformation to high grade is associated with a lower survival rate and a higher rate of distant metastases.^7^, ^8^ The FDG PET/CT would be of great help to determine the primitive tumor and to look for metastases. However, it has not been made due to its unavailability. Surgery is the mainstay of treatment of salivary gland ACC. No prospective randomized studies were conducted to evaluate the efficacy of adjuvant therapy with radiotherapy (RT) or chemotherapy.^9^ Some authors have proposed multiple indications for postoperative RT such as: positive surgical margins, tumor adjacent to the facial nerve, lymph node metastases, extraparotid extension, and tumors greater than 4 cm.^9^, ^10^ Others have suggested that postoperative RT indications would be the presence of high‐grade histologic features (signs of lympho‐vascular invasion and elevated mitotic rates) and positive resection margins.^9^, ^11^

Local recurrence rates are around 20%, while 10% of patients may develop metastatic disease.^12^

Chemotherapy is indicated for metastatic tumors but, given the rarity of this entity, there is no consensus or solid recommendations regarding the chemotherapy protocols that should be administered.^12^ In the case of salivary glandular ACC, chemotherapy protocols are extrapolated from other metastatic head and neck malignancies. Combination therapy has provided better response rates than monotherapy.^13^ Retrospective studies showed that the best overall response rate (46%) was obtained with a chemotherapy regimen including cyclophosphamide, Adriamycin, and Cisplatin.^12^, ^13^ In the case of primary pancreatic tumor, 5‐FluoroUracil (5‐FU) and Gemcitabin are the most common chemotherapeutic agents used in previous case reports of ACC of the pancreas.^14^

However, in our case report, the primary tumor was not known; and all the explorations failed to identify the origin of the metastatic ACC. Chemotherapy protocols based on taxane, carboplatin, fluorouracil, cyclophosphamide, and epirubicin were all ineffective.

The prognosis of ACC is variable from many series studies, with 10‐ and 20‐year survivals of 88% and 83%, respectively, in the literature.^9^, ^15^ Poor survival in our patient may be explained by late diagnosis, metastatic stage at diagnosis, soft tissue metastases, and unknown primary.

The management of metastatic ACC is a real challenge. Large prospective studies are needed to establish the best therapeutic strategy and select the most effective cytotoxic drugs for the treatment of this rare entity.

## CONFLICT OF INTEREST

All authors declare that they have no conflict of interest.

## AUTHOR CONTRIBUTIONS

IW: involved in acquisition of data, analysis and interpretation of data, drafting of the manuscript, and critical revision of the manuscript for important intellectual content. IB: involved in analysis and interpretation of data, drafting of the manuscript, and critical revision of the manuscript for important intellectual content. SM: involved in analysis and interpretation of data, drafting of the manuscript, and critical revision of the manuscript for important intellectual content. EF: involved in drafting of the manuscript and critical revision of the manuscript for important intellectual content. CI: involved in drafting of the manuscript and critical revision of the manuscript for important intellectual content. AN: involved in drafting of the manuscript and critical revision of the manuscript for important intellectual content. HM: involved in drafting of the manuscript and critical revision of the manuscript for important intellectual content. BFL: involved in critical revision of the manuscript for important intellectual content. MM: involved in critical revision of the manuscript for important intellectual content. BAS: involved in critical revision of the manuscript for important intellectual content.

## ETHICAL APPROVAL

This study is exempt from ethical approval in this institution.

## CONSENT

Consent has been obtained from the patient.
